# Evolutionary history and colonization patterns of the wing dimorphic grasshopper *Dichroplus vittatus* in two Argentinean biomes

**DOI:** 10.1038/s41598-022-05162-6

**Published:** 2022-02-21

**Authors:** Natalia Rosetti, Daniela Krohling, Maria Isabel Remis

**Affiliations:** 1 Departamento de Ecología, Genética y Evolución (DEGE), Facultad de Ciencias Exactas y Naturales, Instituto de Ecología, Genética y Evolución de Buenos Aires (IEGEBA) (CONICET-UBA) Intendente Güiraldes 2160, C.A.B.A., Universidad de Buenos Aires, C1428EGA Buenos Aires, Argentina; 2 CONICET & FICH-UNL (Universidad Nacional del Litoral), CC 217, S3001XAI, Santa Fe, Argentina

**Keywords:** Genetics, Population genetics

## Abstract

Quaternary climate oscillations and modification of the environment by humans have played an important role in shaping species distribution and genetic structure of modern species. Here, population genetic parameters were inferred from the analysis of 168 individuals belonging to 11 populations of the South American grasshopper, *Dichroplus vittatus*, distributed in two Argentinean Biomes (Grassland and Savanna), by sequencing a 543 bp of the mitochondrial COI gene. Overall, we detected considerable haplotype diversity and low nucleotide diversity. AMOVA analyses showed a significant degree of differentiation among Biomes and between populations. Two major mitochondrial lineages can be distinguished. The haplogroup containing the most common haplotype split 17,000 years BP while the haplogroup including the second most common haplotype has a divergence date of about 11,700 years. Approximate Bayesian Computation (ABC) analyses showed that the palaeodemographic scenario that best fitted our data is consistent with a hypothesis of divergence from an ancestral population and subsequent admixture with Grassland-Savanna (South–North) direction. Our results suggest that populations located in both Biomes would derive from a single ancestral population that colonized the region after the Last Glacial Maximum and Grassland would have a more ancestral origin than Savanna. Further, our results emphasize the importance of human-mediated dispersal in the reconfiguration of genetic diversity of species with potential pest capacity.

## Introduction

The environment of South America changed dramatically between glacial and interglacial intervals (ca. 100 ka. cycles for most of the Quaternary) in response to natural fluctuations in the Earth’s physical system^1^. Climate change, such as Quaternary oscillations, as well as dispersal are two major factors determining the contemporary distribution and genetic diversity of organisms^1,2^. Demographic events, such as stepping-stone expansion^3^, human-related dispersal^4^ and active migration^5,6^ of species, blurred their distribution ranges^7^.

Genetic methods are widely applied to test phylogeographic hypotheses on Quaternary population dynamics, glacial/interglacial fluctuations, glacial refugia and postglacial recolonization^7–9^. The places where species persist during glaciations have generally been described as refugia^10^.The way in which species are isolated within such refugia, and the timing and mode of expansion from them after the improvement of environmental conditions, have become highly important topics to get deeper insight into the evolutionary processes that shaped the current genetic diversity.

Pleistocene events, mainly the Last Glacial Maximum (LGM- 26,000 to 19,000 years before present; 26–19 ka BP) are thought to have affected the species range and the distribution of genetic variation among current populations of South America even in non-glaciated regions^11,12^.

Biogeographical approaches consider the Andes as a driver of species diversification; however, historical linkages among South American biomes are still under examination^13^. Particularly, there are few studies on the biogeography and species diversification in the tropical and temperate plains of southern South American grasslands^14,15^. Some papers proposed survival areas during glaciation periods in species distributed in non-glaciated Argentine regions far away from Central Argentina^16–19^. Particularly, there are not many examples analyzing the effect of past climatic events in Central Argentina. The most relevant report proposed that this region is climatic and demographically unstable for an endemic shrub^17^.

In Central Argentina converged the Temperate Grasslands, Savannas and Shrublands (TeGSS) and the Tropical and Subtropical Grasslands, Savannas and Shrublands (TrSGSS) biomes (according to^20^). In South America TeGSS, also known as “Grassland”, include Pampean Prairies, Gramineous Steppe and Patagonian Mallines whereas the TrSGSS, belongs to the southern edge of the region well known as “Savanna”, include the Savannah itself, the Park, and the Shrub Steppe^21^.

Between the natural quaternary regions comprising the central area of Argentina, and described by Iriondo^22^ by taking account of their geological characteristics, the Pampas plain is the most extensive. It covers the lowlands from the Atlantic coast and the Paraná River (east) to the Andean piedmont (west), and between the Gran Chaco (north) and Patagonia (south). The Pampean Ranges are located in the easternmost part of the Central Andes, formed by elongated mountain chains that rise sharply from the surroundings and alternate with narrow valleys and mud-flats, predominantly trending north–south.

Iriondo & García^23^ and Clapperton^24^ indicated for the LGM a generalized advance of glaciers in the Andes Cordillera and South Patagonia and a widespread aridity in the lowlands. After that, many authors^25–29^ based on multi-proxy climate reconstructions inferred extensive dry conditions for the Pampas plain during the LGM.

In the South Pampa plain, the cold desert environment of the LGM is represented by the development of dune fields (Pampean Sand Sea), formed by winds of SW–NE and S–N directions. In the North Pampa, the climate during the LGM was peridesertic, promoting sedimentation of windblown dust in a steppe environment to form a loess belt^25,30^. Iriondo & García^23^ estimated for the LGM a shifting of the present Patagonian climatic province of ca. 750 km to the NE of their present position, covering the entire Pampa plain. An important influence on conditions over Patagonia and the adjacent Pampas during the LGM was the growth of a large conterminous ice cap over de Southern Andes, increasing the strength and consistency of anticyclonic circulation and generating strong drying (cold) winds from the SW^23,24^. Geological proxy data suggest a northward shift (up to 33°S) of the Westerlies^31^.

The analysis of new examples can improve our understanding of how species have responded to past climate changes, and where they endured periods of adverse climates in Central Argentina, forecasting how current climate change may affect species^10^. Some species of Orthoptera are distributed in Central Argentina and constitute good models to analyze this issue.

Orthoptera species of the Acrididae family are an essential component of both, healthy, and disturbed ecosystems. As primary consumers, they are important from a nutrient cycling perspective in ecosystem^32–34^.

Some species of Acridids may be also considered of economic interest due to their ability of colonize modified environments and later experience a demographic expansion in their distribution range causing damage to crop and even to other components of biological diversity^35^. The genus *Dichroplus* included 18 species which are frequently grassland residents and are considered of economic importance because of the damages they cause to crops and pastures^36–38^.

*Dichroplus vittatus* is an especially harmful species of this genus. A South American nonselective polyphagous grasshopper with agronomic importance in Argentina, it is distinctive since in natural populations it may display wing size polymorphism^38–41^.

In fact, in *D.vittatus* a poorly developed wing morph (brachypterous) can coexist with a flight-capable morph with fully developed wings (macropterous) in populations from Central-West Argentina^41^.

Recent analyses indicate that dimorphic populations of *D. vittatus* have differences in their dispersal capacity. Populations with high frequency of long-winged individuals (macropterous) would show greater colonization potential revealing information for possible implementation of pest management plans^41^. To date, there are neither studies about genetic differentiation, nor about demographic and phylogenetic history of this species.

The present contribution constitutes the first attempt to analyze the evolutionary history and colonization pattern of *D.vittatus* in populations from Central Argentina that belong to two Biomes (according to Olson^20^; Costa^21^) by using sequences of mitochondrial DNA. We analyzed populations from the northwestern border of the Grassland Biome (TeGSS) (corresponding mainly to South Pampa quaternary region) and in the southwestern edge of the Savanna Biome (TrSGSS) (corresponding to Inter-mountain valleys of the Pampean Ranges region) (according to the classification of Iriondo^22^, which represents a fraction of the species distribution.

The particular aims are (1) to infer genetic diversity (2) to estimate population structure (3) to analyze relationships and divergence among lineages (4) to explore historical demography and its association with Quaternary glacial or interglacial periods (5) to infer which hypothesis best explains the evolutionary history of the species in the studied region.

Our analysis will help to determine if the populations located in the studied area derived from multiple or a single glacial refuge and identify the historical colonization routes and the demographic or historical events that shaped the genetic diversity observed today in this species.

## Results

### Genetic diversity and population structure

**Figure 1:**
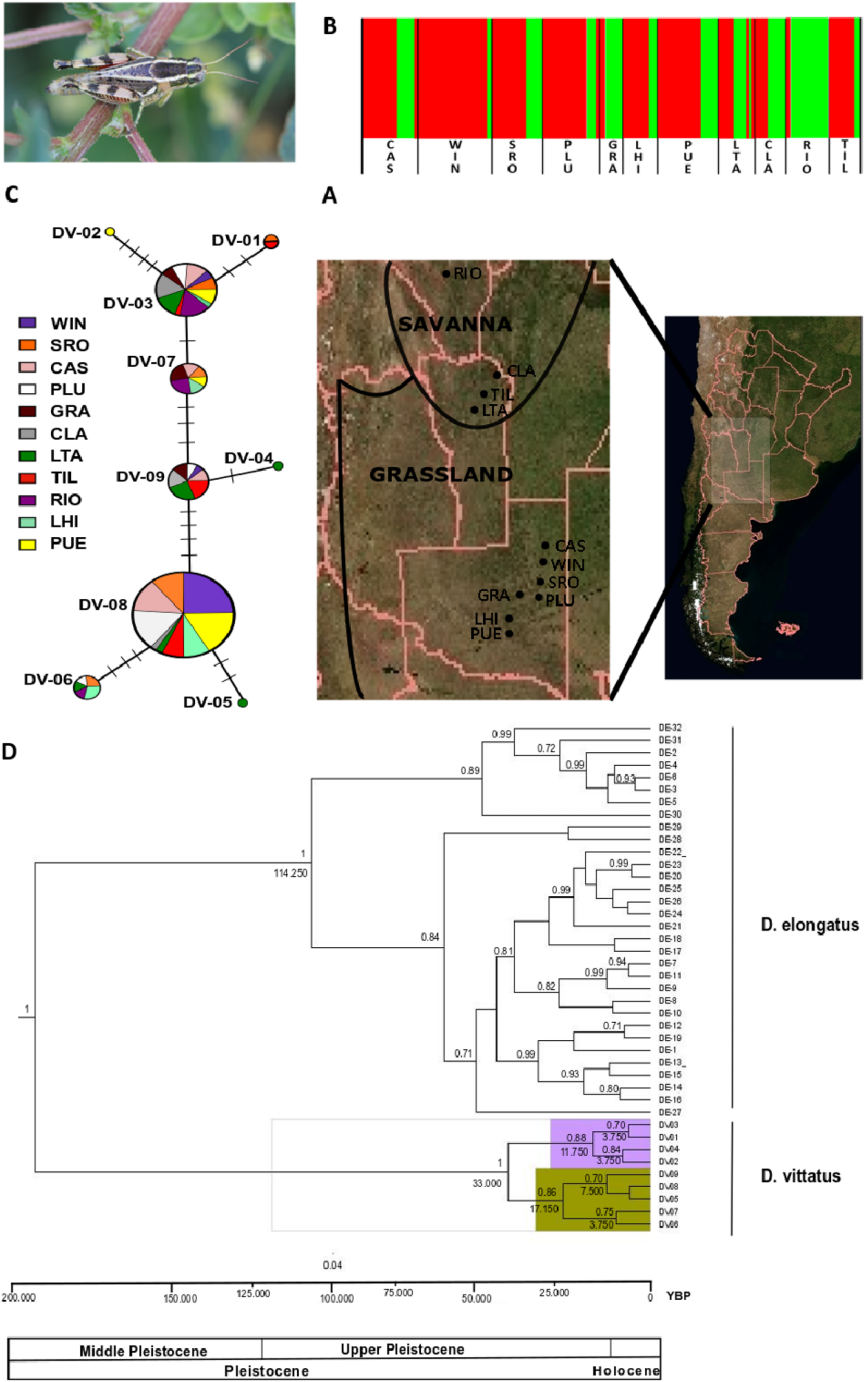
(**A**) Geographic distribution of 11 populations of *D. vittatus* located in two Argentinean Biomes (Grassland and Savanna). The map was extracted from https://www.gifex.com/America-del-Sur/Argentina/Satelitales.html and edited with free open Inkscape 1.1 software (https://inkscape.org). (**B**) Bayesian clustering assignment of individuals analysis implemented in the program BAPS under mixture of groups of populations model based on mitochondrial genetic data. The bar plot shows the group assignments of 167 individuals for K = 2 (the optimal number of clusters). The vertical black lines separate populations. (**C**) Median-Joining network for the COI mtDNA haplotypes of *D. vittatus*. Each circle represents a haplotype, and circle size is proportional to haplotype frequency. Colors indicate the proportion of individuals sampled in different populations within the study area. Branch lengths are proportional to the number of substitutions per nucleotide site. (**D**) Mitochondrial haplotypes tree chronogram estimated by BEAST for *D. vittatus* with divergence date estimates for well supported nodes below and posterior probability above nodes. The mean age is given in years. A geological time scale is shown at the bottom. *D. elongatus* is used as outgroup.

A 543 bp fragment of the mitochondrial gene COI was sequenced from 168 samples of *D.vittatus* from 11 populations distributed in Central-West Argentina (Fig. 1). Nucleotide substitutions defined 9 haplotypes from 5 polymorphic sites, 3 of which were singletons and 6 were detected more than twice. Savanna and Grassland Biomes shared 6 haplotypes. Savanna showed 2 private haplotypes, both from LTA population, whereas in Grassland 1 private haplotype was identified in PUE population. The most frequent haplotype in Grassland was DV08, however Savanna exhibited DV03 as the most frequent haplotype.

Considerable haplotype diversities were detected whereas nucleotide diversities proved to be low (Table 1). Savanna showed higher genetic diversity with respect to Grassland, being LTA the most diverse population. In Grassland, GRA and SRO populations were the most diverse ones while WIN had the lowest values of genetic diversity (Table 1).

**Table 1:** Summary of haplotype frequencies, genetic diversity indices (haplotype diversity (h) and nucleotide diversity (π) with their standard deviation) and incidence of fully developed wing morph (M) for 11 population of *D. vittatus* distributed in two Biomes (Grassland and Savanna).

Grassland								Savanna			
Haplotype	WIN	SRO	CAS	PLU	GRA	LHI	PUE	CLA	LTA	TIL	RIO
DV-01		1								1	
DV-02							1				
DV-03	2	3	4	3	2	1	3	6	5	1	6
DV-04									1		
DV-05									1		
DV-06		2		1		2			1		1
DV-07		2	2		3	2	1				3
DV-08	23	10	11	14		7	15	2	2	6	1
DV-09	1		2	1	2			3	4	3	
N	26	18	19	19	7	12	20	11	14	11	11
M*	0.75	0.32	0.06	0.11	0	0	0	0.21	0	0	0.1
h	0.218 (0.103)	0.652 (0.105)	0.632 (0.101)	0.450 (0.128)	0.762 (0.115)	0.651 (0.132)	0.432 (0.126)	0.655 (0.111)	0.813 (0.073)	0.673 (0.123)	0.673 (0.123)
π	0.0009 (0.0008)	0.0027 (0.0019)	0.0024 (0.0017)	0.0018 (0.0014)	0.0017 (0.0015)	0.0020 (0.0016)	0.0023 (0.0016)	0.0026 (0.0019)	0.0030 (0.0021)	0.0025 (0.0019)	0.0019 (0.0015)
h	0.537 (0.049)							0.775 (0.028)			
π	0.0021 (0.002)							0.0029 (0.002)			

*Data from Rosetti and Remis^41^.

Hierarchical AMOVA analyses were performed with the populations grouped by their geographical distributions considering the Biome to which they belong (Table 2). AMOVA using Ф_ST_ accounting for frequencies and divergence between haplotypes showed that 15.8% of the genetic variance was found among Biomes (Ф_CT_ = 0.158, *P* = 0.003), differences among populations within Biomes accounted for 8.28% (Ф_SC_ = 0.098, *P* = 0.007) of the total variation, while 75.91% of the variance could be attributed to within-population variability (Ф_ST_ = 0.240, *P* = 10^−5^). AMOVA based on F_ST_ statistics revealed that 15.7% of genetic variation is distributed among Biomes (F_CT_ = 0.158, *P* = 0.003), while 7.4% of variance is explained by differences among populations within Biomes (F_SC_ = 0.08, *P* = 0.005) and 76.8% corresponded to differences between individuals within populations (F_ST_ = 0.23, *P* = 0.003) (Table 2). AMOVAs for each Biome showed that all variance components were significant and that partitioning of molecular variance was similar in both regions (Table 2).

**Table 2:** Analyses of molecular variance (AMOVAs) based on F_ST_ and φ_ST_ values for 11 populations of *D. vittatus* distributed in two Biomes.

		df	F_ST_	SS	% of variance	*P* values	Φ_ST_	SS	% of variance	*P* value
	Among Biomes	1	0.158	4.492	15.78	**0.003**	0.158	9.535	15.81	**0.003**
	Among population within Biomes	9	0.088	6.129	7.38	**0.005**	0.098	13.733	8.28	**0.007**
	Within populations	157	0.232	43.445	76.85	**0.003**	0.240	90.089	75.91	**10**^**–5**^
	Total	167	-	54.065	100	-	-	113.357	100	-
Grassland	Among populations	6	0.087	3.882	8.71	**10**^**–3**^	0.093	8.670	9.28	**0.005**
	Within populations	114	-	28.159	91.29	-	-	60.173	90.72	-
Savanna	Among populations	3	0.086	2.246	8.64	**0.027**	0.109	5.063	10.86	**0.032**
	Within populations	43	-	15.286	91.36	-	-	29.916	89.14	-

df: degree of freedom; SS: sum of squares; *p*-value: level of statistical significance.

Statistically significant and higly significant results are shown in bold type.

BAPS analysis of the mtDNA locus defined two genetic clusters (lnL = − 8303.607) (Fig. 1B), although, evidenced of strong pattern of admixture is observed. In general, all Grassland (except GRA) and Savanna (expect TIL) populations proved to have similar genetic constitution which may be explained by gene flow or shared ancestral variation.

### Phylogenetic analysis, divergence time and haplotype relationship

The median joining network illustrating the relationship of haplotypes indicated two haplotype lineages, with the overall network characterized by irregular clustering of haplotypes by population or Biome (Fig. 1C). The most frequent haplotypes (DV08 and DV03) appeared as central haplotypes, from which several less frequent haplotypes derived by at most four mutational steps, evidencing the ancestral condition of these haplotypes. Some star-like connections were found in different parts of the network, suggesting some level of phylogeographic structuring, suggesting a relatively recent expansion in size.

To infer relationships and divergence date between *D.vittatus* haplotypes, we implement a Bayesian approach through BEAST software considering *D.elongatus* haplotypes as outgroup. The BI tree showed strongly significant support for the divergence between haplotypes grouped by species (Fig. 1D). Two major mitochondrial lineages can be distinguished in *D.vitttatus*, which seem to have diverged 33,000 years BP (33 ka BP) (95%CI: 17,000–53,000) during the Upper Pleistocene. The haplogroup containing the most common haplotype (DV08) split 17,000 years BP (95%CI: 7000–41,000) while the haplogroup including the second most common haplotype (DV03) has a divergence date of about 11,700 years (95%CI: 6000–18,000) (Fig. 1D).

Population history molecular clock analyses inferred from BEAST suggested that all populations, both from Savanna and Grassland, showed a temporally uniform origin, TMRCAs ragging from 15,000 to 17,000 years BP (Table 3), placing the origin of all studied populations after the Last Glacial Maximum (LGM), which concluded with the Late Pleistocene glacial retreat.

**Table 3:** Bayesian coalescent estimation of time to most recent common ancestor (TMRCA) among *D.vittatus* populations with 95% highest posterior density modeled assuming a relaxed molecular clock as implemented in BEAST.

Biome	Population	TMRCA	95% credibility interval
Grasslands	CAS	0.017	8.5 E-4–0.047
	WIN	0.016	8.4 E-4–0.047
	SRO	0.017	7.7 E-4–0.043
	PLU	0.017	7.7 E-4–0.047
	GRA	0.015	4.8 E-4–0.044
	LHI	0.017	8.8 E-4–0.047
	PUE	0.016	5.9 E-4–0.046
Savanna	CLA	0.016	8.6 E-4–0.047
	TIL	0.016	8.4 E-4–0.046
	LTA	0.017	9.0 E-4–0.046
	RIO	0.017	8.2 E-4–0.048
Grasslands		0.019	3.5 E-3–0.045
Savanna		0.020	3.2 E-3–0.045

Times of divergence among populations are in million years.

### Demographic inference

**Figure 2:**
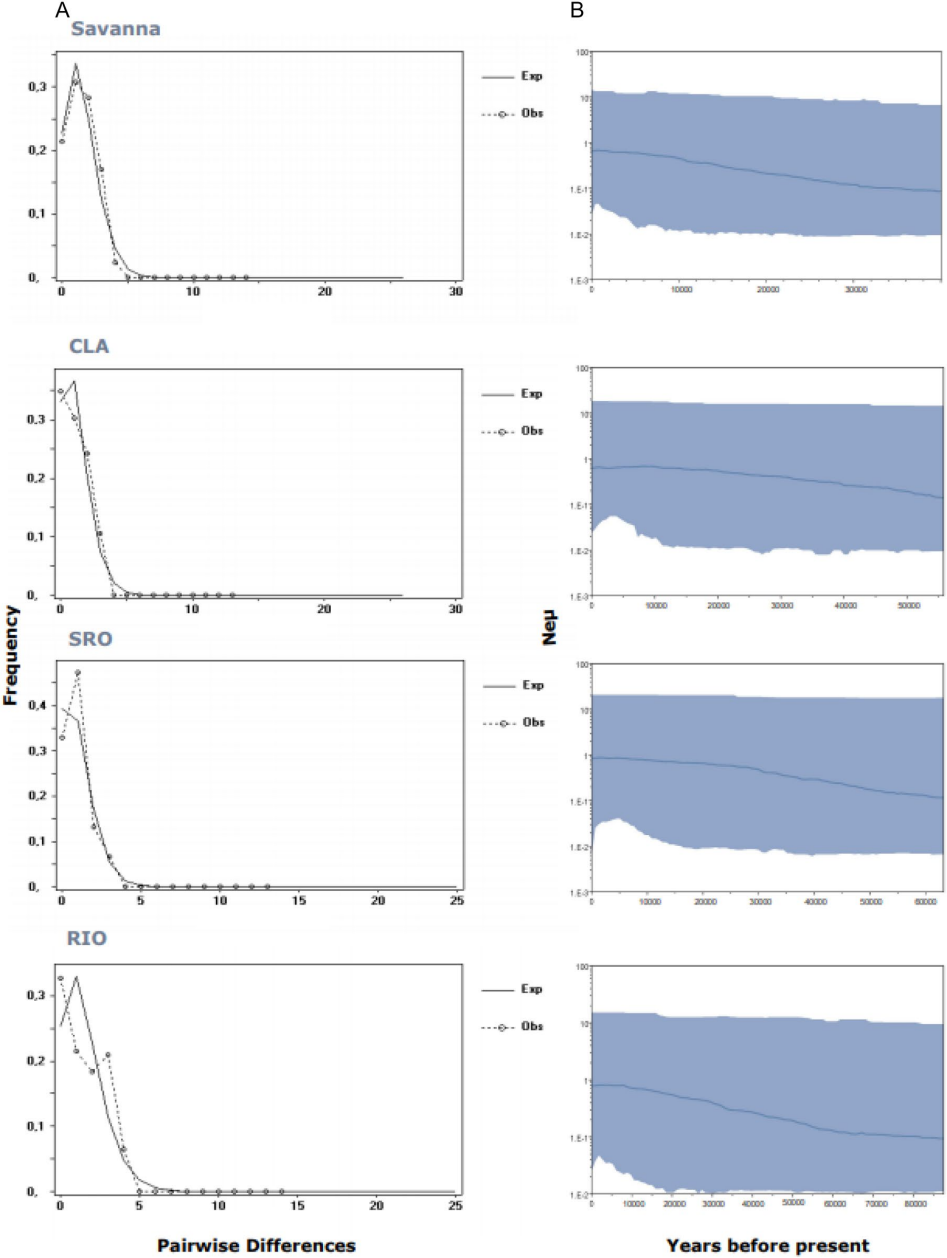
Mismatch distribution (**A**) and Bayesian skyline plots (**B**) of the Biome Savanna and the populations that showed significant raggedness index (r). For the mismatch distributions, solid lines show observed frequency distribution while dashes lines show the distribution expected under the sudden-expansion model. For the Skyline analysis, the x-axis represents time in units of years and the y-axis represents effective population size as Ne on a log scale. The blue line depicts the median population size, and the shaded areas represent the 95% highest posterior density intervals.

Neutrality tests were performed to detect signs of recent population expansion. Fu’s Fs value was negative and non-significant for both Biomes, whereas Tajima’s D test showed positive but non-significant values in both cases (Table 4). The mismatch distribution for Savanna showed a relatively smooth, unimodal observed distribution that closely matched the expected distribution under an exponential growth rate model (Fig. 2A) and raggedness indice was consistent with a recent population expansion (Table 4). The mismatch distribution of Grassland was roughly multimodal (not shown) and did not show significant departures from an equilibrium model (Table 4), a pattern indicative of more deeply diverging lineages.

**Table 4:** Demographic summary statistics Tajima’s D and Fu’s FS and mismatch distribution raggedness index (r) based on mitochondrial COI sequence data of 11 populations of *D. vittatus* located along two Argentinian Biomes.

Biome	Population	Tajima’s D		Fus’s Fs		Mismatch distribution	
		D	p	Fs	p	r	p
Grassland	WIN	− 0.869	≥ 0.05	− 0.002	≥ 0.05	0.547	≥ 0.05
	SRO	0.793	≥ 0.05	− 0.282	≥ 0.05	**0.039**	**0.02**
	CAS	1.467	≥ 0.05	0.595	≥ 0.05	0.102	≥ 0.05
	PLU	0.393	≥ 0.05	− 0.105	≥ 0.05	0.266	≥ 0.05
	GRA	0.687	≥ 0.05	− 0.056	≥ 0.05	0.292	≥ 0.05
	LHI	0.372	≥ 0.05	− 0.404	≥ 0.05	0.035	≥ 0.05
	PUE	0.254	≥ 0.05	0.474	≥ 0.05	0.671	≥ 0.05
	Total	0.500	≥ 0.05	− 0.528	≥ 0.05	0.122	≥ 0.05
Savanna	CLA	1.316	≥ 0.05	1.022	≥ 0.05	**0.163**	**0.04**
	LTA	0.994	≥ 0.05	− 1.511	≥ 0.05	0.107	≥ 0.05
	TIL	0.043	≥ 0.05	− 0.055	≥ 0.05	0.112	≥ 0.05
	RIO	− 0.031	≥ 0.05	− 0.569	≥ 0.05	**0.145**	**0.03**
	Total	1.471	≥ 0.05	− 1.548	≥ 0.05	**0.044**	**0.05**

Significant values are highlighted in bold.

Analyses at population level did not detect Tajima’s D or Fu’s Fs significant coefficients (Table 4). In Grassland, mismatch distribution analysis showed multimodal pattern in most populations, suggesting constant population size, sustained subdivision, or both, for a long period of time, except for SRO population. Harpending’s raggedness index (r) suggested longer periods of population stability for WIN and PUE (Table 4). SRO population showed a unimodal distribution compatible with a recent expansion event (Fig. 2A) and a Harpending’s raggedness index (r) supporting a significant apart of population stability (Table 4). In Savanna, mismatch distributions appeared to be unimodal for CLA and RIO, closely matching the expected distribution under the sudden expansion model (Fig. 2A). In agreement, Harpending’s raggedness index (r) reject the hypothesis of population stability (Table 4).

Although neutrality test indexes and mismatch distributions can provide insights into whether population growth has been expansive, they are not able to provide information about the shape of population growth over time. Therefore, to estimate the shape of effective population size (Ne) change through time we constructed Bayesian skyline plots (BSPs). The demographic scenario for Savanna presented by the BSP analyses showed a dual pattern, a short time of constant population size, followed by a long period of constant demographic expansion estimated to have started between 23,000 and 20,000 years BP (Fig. 2B). Grassland BSP showed a stable population effective size period finishing 20,000 years BP, followed by approximately 19,500 years of demographic expansion. Since 450 years BP Grassland remained at a stable population size (Fig. S1). This analysis showed that both Biomes began a process of expansion after the last Pleistocene glaciation, maintaining relatively stable population sizes before and during the LGM. The BSP for CLA and RIO (populations of Savanna with significant recent expansion pattern assessed by the mismatch distribution) recovered signs of constant population size growth being stronger 10,000 years BP close to the beginning of the Holocene. SRO the only Grassland population showing recent size growth, according to the mismatch distribution analysis, showed a BSP consistent with a population size increase approximately 16,000 years BP (Fig. 2B). The width of the credibility intervals represents some level of both, phylogeographic and coalescent uncertainty.

### Historic models and evolutionary inference

Coalescent estimates of effective migration rates revealed a complex pattern of asymmetrical gene flow among Biomes (Table 5). The estimates of historical gene flow based on both coalescence and conventional F_ST_ approaches demonstrated significant number migrants per generation (greater than 1) between regions belonging to different Biomes (Table 5). The analysis based on coalescence revealed asymmetrical gene flow in a south-north direction. An important ancestral dispersion from Grassland to the Savanna region with an average number of historical migrants per generation (N_e_m_f_) of 7.42 whereas gene flow in the opposite direction was 2.77 (N_e_m_f_).

**Table 5:** Most probable estimates of migrants per generation between local populations of *D.vittatus* belonging to Savanna and Grassland Biomes based on Bayesian (Nem_ij_) and traditional FST approaches (Nm) between local areas belonging to Savanna and Grassland Biomes in Argentina.

	Nem12	Nem21	95% CI	FST	Nm
(1) Grassland	7.42		1.63–21.00	0.17	2.41
(2) Savanna		2.77	1.60–8.36		

The demographic reconstruction resulting from the ABC analysis identified scenario 1 (Divergence and subsequent admixture with South-North direction hypothesis) as the most highly supported event to explain the current phylogeographical structure of *D. vittatus* (Table 6). Both the average relative bias and the RMSE were low and did not indicate any systematic over-or underestimation of the various parameters. Model checking performed through a PCA of posterior distributions of scenarios showed a good placement of the observed dataset in the posterior probability cloud (Fig. S2).

**Table 6:**
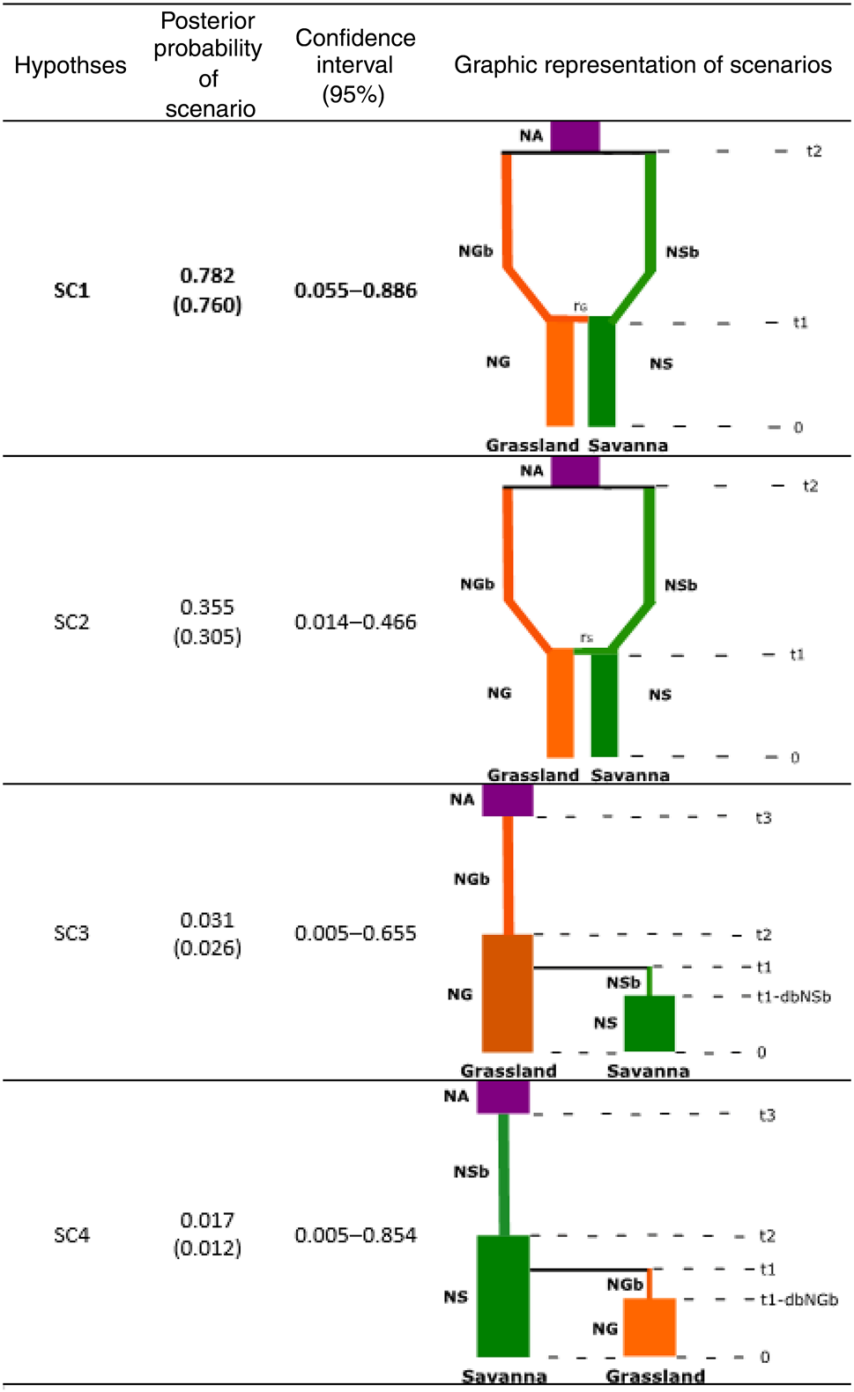
ABC comparison between the hypothesized evolutionary scenarios.

Bayesian posterior probabilities (direct and logistic regression in brackets) approaches, 95% credible interval (95% CI), and schematic representation of each tested scenario estimated using DIYABC for *D. vittatus* populations located in two Argentinean Biomes. The most probable scenario is highlighted in bold letters. NA: effective size of ancestral population, NS: effective size of Savanna, NG: effective size of Grassland, NGb: effective size of Grassland prior to population increase, NSb: effective size of Savanna prior to population increase, t: time in number of generations (generation time in *D. vittatus* is estimated in 1 year), r: admixture rate. Bar widths in the schematic representations of models are proportional to effective population size (indicating reductions and expansions ranging of the previous size). Gray bars represent divergence events.

The type I error indicates that the probability of excluding the best scenario (SC1) when it is the correct one is low (0.043, 0.046 direct and logistic approaches respectively), and the type II error indicates that the probability of selecting the best scenario when it is not the correct one is also relatively low (0.027 and 0.016, direct and logistic values respectively). Posterior distribution of parameters for SC1 and RMAE values are shown in Table 7. This scenario assumes that populations settled in Grassland and Savanna diverged 21,000 years BP from an ancestral population that could be part of a glacial refuge. Effective population size estimates suggest that the ancestral source of colonizers was 6,750 individuals. Both Grassland and Savanna population size after funding event was reduced, showing a mean value of 5510 and 4660 respectively. Later, and very recently (181 years BP) an admixture event of both Biomes occurs through gene flow in a Grassland-Savanna direction, with an admixture rate of 0.550. This lead to a recent increase of population size from Grassland (Ne = 8540) and Savanna (Ne = 7870).

**Table 7:** Model parameters estimated (mean, median and mode with 95% confidence intervals and RMAE values (relative median of the absolute error)) from posterior distribution of the best evolution scenario (SC1).

Parameter	Mean	Median	Mode	q025	q975	RMAE
NA: Ancestral population size	6750	7130	9380	3820	9830	0.163(0.265)
N1 (NG): Effective size of Grassland	8540	8820	9900	6470	9950	0.161(0.209)
N3 (NGb): Population effective size of Grassland after colonization from the ancestral population	5510	5840	6690	3120	8280	0.177(0.257)
N2 (NS): Effective size of Savanna	7870	7880	8790	5605	9070	0.173(0.210)
N4 (NSb): Population effective size of Savanna after colonization from ancestral population	4660	4780	5860	3110	8740	0.126(0.288)
t2: time to divergence of Biomes from ancestral population	21,100	19,200	11,700	6320	60,600	1.223(2.760)
t1: time of admixture event among Biomes	181	199	89.4	33.4	392	3.930(25.538)
r3: admixture rate	0.550	0.260	0.082	0.039	0.830	0.276(0.587)

RMAEs are low, indicating that parameter estimates are reliable. As usual with demographic analyses, confidence intervals are wide however, broad intervals are mostly attributable to the limited information contained in the dataset rather than inaccurate choice of the priors or model misfit, as confirmed by model checking and RMAE values.

## Discussion

Historical events, particularly Pleistocene glacial/interglacial cycles, have had a critical impact on generating phylogeographic structure in species^7^. The atmospheric circulation during the Upper Quaternary displayed patterns that differed significantly between glacial and interglacial periods. These differences may have been similar to those that existed between the LGM and a warm period represented by the mid-Holocene *Climatic Optimum*^42^. Particularly, the LGM is an exciting event for investigating ecosystem responses to climate changes ever since. Paleoenvironmental records suggest that full glacial climates throughout South America (tropical regions comprised) were overall colder than today by about 5 °C^43,44^.

Phylogeographic studies typically examined how species responded to paleoecological events within a single biome or biogeographic region^45–47^. However, some biomes have been more influenced by glacial impact than others. Here we report how a study of the wing dimorphic grasshopper *D. vittatus*, performed on part of the species distribution area across two Biomes of Southern South America (Grassland and Savanna) responded to some extreme climatic events of the Pleistocene, and what are the implications of these historical patterns in the present.

Our results showed genetic differences between both Biomes. The significant population structure can be attributed to differences in topography and precipitations between the temperate (Savanna) and tropical and subtropical (Grassland) regions as previously proposed^17^.

*D. vittatus* exhibited considerable haplotype diversity (h ˃ 0.5) and low nucleotide diversity (π < 0.5%) in Grassland and Savanna Biomes that could be attributed to population growth from a small number of individuals of an ancestral source^48^. The widespread distribution of the most frequent haplotypes (DV08 and DV03) throughout the studied area, as well as the weak phylogeographical signal, also supported the hypothesis of an ancestral expansion by long distance dispersal related to the colonization of isolated populations with very little genetic variation^49,50^. Furthermore, the leading-edge model of post-glacial expansion predicts there will be lower genetic diversity in recently colonized regions^51^. This would support the hypothesis that populations located in both Biomes in the studied area derive from a single ancestral population, since if there had been more than one; we should expect a higher genetic diversity and exclusive haplotypes in high frequency both in Savanna and in Grassland.

After colonization, the estimates of migration rates suggested significant levels of genetic connectivity between both biomes with predominance of migration from South to North namely from South Pampa (corresponding to Grassland Biome) to Inter-mountain valleys of the Pampean Ranges (corresponding to Savanna Biome). The inferred historical gene flow would indicate some homogeneous environment in both natural quaternary regions in some past periods in the area analyzed here.

Our previous work^41^ suggested that the incidence of the macropterous morph (a proxy of contemporary migration events) is more frequent in populations from Grassland than in the Savanna ones. The frequency of macropterous individuals decreased with longitude and increase with precipitations, indicating that the macropterous morph is more frequent in the humid areas (Pampa plain) of the studied region (mostly populations of the Grassland Biome).

It is traditionally accepted that the frequency of winged and wingless morphs can change through space and over time reflecting a trade-off between dispersal and reproduction in different environments^52–54^. Over time the macropterous frequency can decrease if environment conditions are more suitable (the selective advantage to disperse, decrease) and populations reach incidence of wing morphs like populations in stable habitat in a few generations^53^.

A possible explanation for the current highest incidence of winged morph in Grassland may be that populations from Pampa plain correspond to an area influenced by agriculture and the pattern of wing variation would reflect events of contemporary migration and (re)colonization. Another tentative explanation can be related with different levels of trade-offs between dispersal and reproduction in different environments.

The current gene flow (inferred by the incidence of macropterous morphs) from Grassland to Savanna is in line with the detected historic gene flow. However, as mentioned before the incidence of winged forms in addition to environmental factors may also be explained by anthropic activities.

The demographic scenario showed by the Bayesian skyline plots indicated that both biomes experimented a long period of demographic expansion after LGM (started about ca 20 ka BP). The signals of population expansion were also detected through the analysis of mismatch distribution. Savanna’s mismatch analysis showed a distribution expected under a recent population expansion event, being CLA and RIO the populations that experience a clear pattern of expansion. The multimodal mismatch distribution revealed that Grassland represents a relatively stable area, with SRO being the only population showing signs of recent expansion. In general, our demographic studies pointed out Savanna Biome as an unstable area during Upper Pleistocene and Upper Holocene whereas Grassland Biome demonstrated more stability as expected in an oldest area.

Our results evidenced that after ca. 20 ka BP, the two groups, established in Grassland and Savanna, are differentiated from an ancestral population (possibly a refuge or part of a glacial refuge). These results may be in concordance with paleoclimatic evidence. The last deglacial hemicycle, which marked the transition between the last glacial and present interglacial period, was characterized by a general increase in temperature and precipitation in Argentine plains^23,52^. This would have allowed the expansion of *D.vittatus* in the Pampa plain currently represented by the Grassland Biome. Populations were settled later in currently Savanna, developed on the Pampean Ranges Region, perhaps because of a later improvement of environmental conditions.

The inference of population history with DIYABC confirmed that the population that established in Grassland was relatively larger than the Savanna one. In agreement with DIYABC, the two lineages identified by the Bayesian tree are roughly equally represented in the Savanna Biome whereas most of the haplotypes of the Grassland Biome (about 84%) correspond to the oldest lineage. These results support the hypothesis that Grassland would have a more ancient origin than Savanna, either due to higher proximity to the source ancestral population or due to that Grassland area (Southern Pampa) constituted an environment with better postglacial conditions.

The signal of demographic expansion in Savanna is maintained at a slow but constant rate; however, Grassland reached a demographical stability at ca. 450 years BP. It could be related to the cold and arid period known as the Little Ice Age (LIA) occurred in the central region of Argentina between ca. 700–150 years BP^23,55^.

Several works suggested that terrestrial Patagonian taxa and some aquatic species probably survived glacial periods in southern refugia or alternatively recolonized the area from northern latitudes^56–59^. Recent studies revealed insights about the survival areas during glaciation periods in species distributed in non-glaciated Argentine plains^16–18,60^. Lately was proposed a suitable refuge area for populations of an endemic shrub towards the north of the 35° south latitude of Central Argentina during Pleistocene glaciations and a southeast direction of the range expansion during interglaciations^17^.

Another appealing hypothesis supporting the location of the glacial refuge closer to Grassland than Savanna would assume a micro-refuge located East of Grassland close to the Atlantic Rainforest Ecoregion (in Southern Brazil) where the existence of glacial refuges in general and in particular for insects is known^16,61–63^. It can be hypothesized that the founders of the populations found today in the studied area of Grassland, could come from peripheral refuges or micro-refuges of the Atlantic Rainforest Ecoregion, since, the results show that populations were established in the studied Grassland area in a short term after the LGM and on the other hand, low general genetic diversity, widespread distribution of frequent haplotypes and weak phylogeographic signal indicates colonization by long distance dispersal from an ancestral population of low genetic diversity and effective size, compatible with the characteristics of a peripheral refuge.

However, we studied only part of the geographic range of this species and further phylogeographic studies analyzing other geographic areas of this wide distributed species in Argentina may improve our knowledge about it evolutive history.

Additionally, we detected an admixture event with Grassland-Savanna direction about 185 years ago. The admixture occurs due to gene flow of individuals that leave from Grassland and enter in the Savanna Biome (which is also confirmed by the Migrate analysis). However, it turned out not to be a colonization process where individuals from Grassland totally or partially replaced those of Savanna; rather, a mixture of individuals occurred, what can also be seen in the BAPS analysis and through the haplotype network. It could be related to the climatic phase very well described by Darwin in his historical voyage around the world as “the Great Drought”, that was interpreted by Iriondo & Kröhling^64^, as a short late occurrence of the LIA climate, which was characterized by a marked dryness in the Pampas. The Great Drought comprises part of the first half of the nineteenth century (AD 1800/1810 and AD 1827/183239). During most of the LIA, the Pampa plain registered an increasing demographic change after the Spanish conquer and the development of agricultural practices.

Patterns of genetic structure can change due to species dispersal ability frequently associated with environmental conditions^64^. Human-mediated dispersal can reshape the genetic structure of animal populations and superimpose new signatures on existing natural phylogeographical patterns. Humans fundamentally affect dispersal, directly by transporting individuals and indirectly by altering landscapes. This human-mediated dispersal (HMD) modifies long-distance dispersal, changes dispersal paths, and above all benefits certain species or genotypes while disadvantaging others^65^.

Our results would indicate that there were two mayor demographic processes in the last 20 ka for the studied distribution of *D. vittatus*. One was the establishment of populations in Grassland and Savanna from a single glacial refuge located probably in the vicinity of the area that currently constitutes the Grassland Biome. This hypothesis is in line with our results which showed that Grassland is composed chiefly by haplotypes from the most ancestral haplogroup, constitutes an area that diverged earlier (approx. 17 ka BP), and it showed to be more stable with larger populations sizes during the last part of the Upper Pleistocene. Savanna Biome was later recolonized from individuals of the same refuge, and it subsequently received migrants from Grassland more recently due to a predominant south-north migration.

On the other hand the mixing event detected by the DYABC analysis in the last 185 years with Grassland-Savanna direction that could be explained through the increase in agricultural exploitation in the Pampas Region, becoming the largest center of crop production in the country during the last decades, which led to the construction of roads and trade routes destined to distribute these products throughout Argentina; this man-mediated dispersal could have influenced the phylogeographic pattern that we see today.

Further studies in other animal models may improve our understanding about the relationships between paleoclimatic changes and phylogeographic patterns in Central Argentina.

## Materials and methods

### Sample collection

A total of 168 adults including both macropterous and brachypterous individuals of *Dichroplus vittatus* were collected from 11 natural populations during the breeding season (January–February; 2011–2013) across two Argentinian Biomes; Temperate Grasslands, Savannas and Shrubland (referred as “Grasslands”) and Tropical and Subtropical Grasslands, Savannas and Shrublands (referred as “Savanna”) biomes (according to Figure 1 of^20^) (Fig. 1A).

### Geomorphological setting

**Figure 3:**
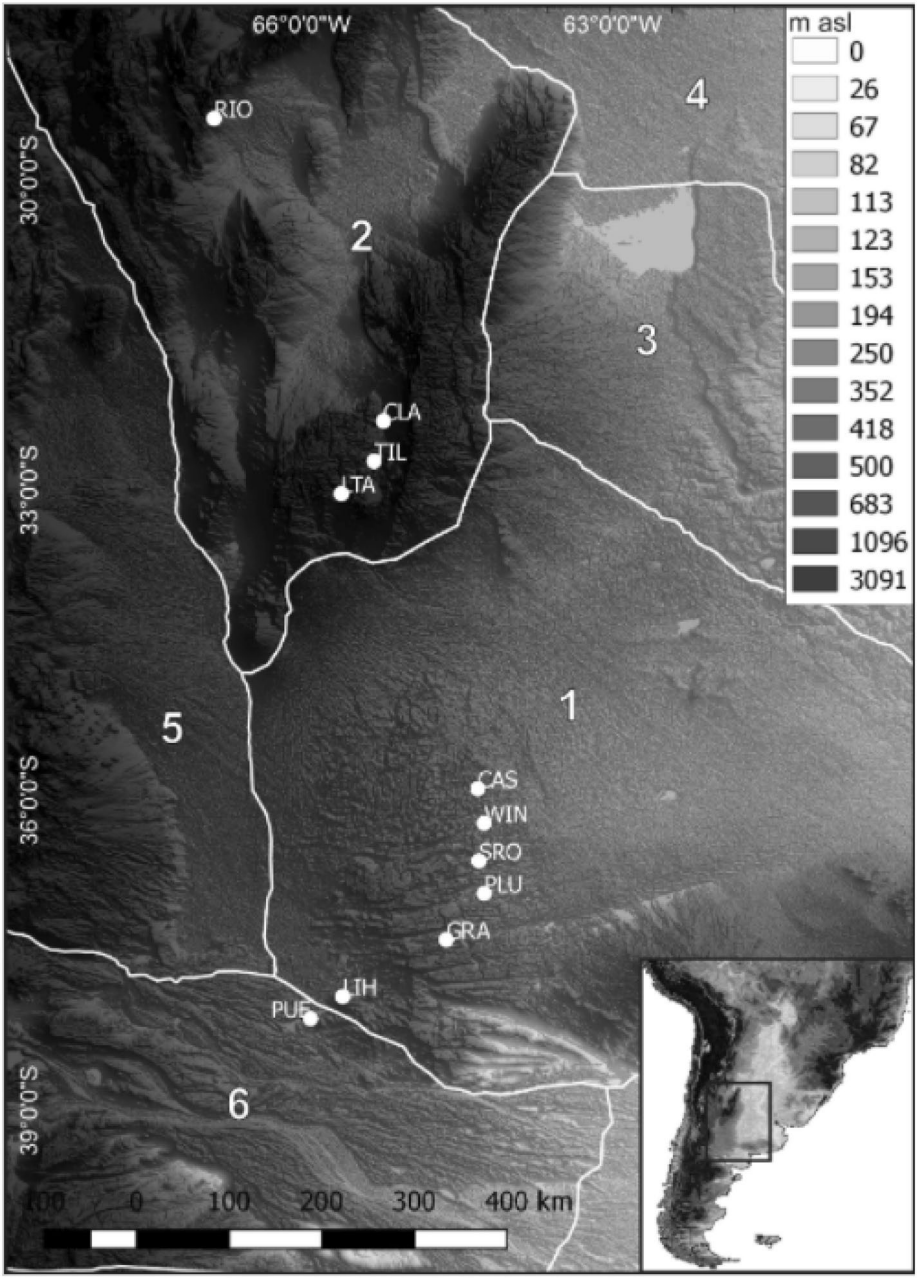
Distribution of the collected samples on a map of the natural quaternary regions of central Argentina taken from Iriondo22. The map is in a Global Multi resolution Terrain Elevation Data (GMTED201093) of 225 m resolution developed by the US Geological Survey (USGS) and the National Geospatial Intelligence Agency (NGA). https://www.usgs.gov/core-science-systems/eros/coastal-changes-and-impacts/gmted2010?qt-science_support_page_related_con=0#qt-science_support_page_related_con. Google Earth Engine© cloud–based platform (GEE; https://earthengine.google.com) and QGIS Geographic Information System (free open source software v.2.18; http://www.qgis.org) were used for accessing and processing the open-source GMTED. Quaternary regions:1. South Pampa. 2. Inter-mountain valleys of the Pampean Ranges. 3. North Pampa. 4. Chaco. 5. Central Andes and the Eastern Piedmont. 6. Patagonia Plateau Region.

Taken account the natural quaternary regions of Argentina discriminated by Iriondo^22^, the collected samples are located in three regions (Fig. 3). The central plain represented by the South Pampa Region contain most of the samples. Aeolian deposits, fluvial fans and belts originated in the Central Andes Cordillera and the piedmont, fluvial paleo-valleys and Ventania and Tandilia Ranges characterize the South Pampa. The Pampean Sand Sea covers most of the South Pampa and includes large longitudinal dissipated dunes, deflation corridors, parabolic dunes and sand sheets. This non-permanent desert body evolved during dry periods of the Upper Pleistocene, also including the LGM^31^. Other samples were collected in the Region of the inter-mountain valleys of the Pampean Ranges^22^, characterized by alluvial fans, salt and playa lakes and dune fields. Only one collected sample is located in the northernmost area represented by a dissected alluvial plain forming part of the Patagonia Plateau Region^22^ (Fig. 3).

### mtDNA amplification and sequencing

We extracted total genomic DNA using the QIAgen DNeasy Blood and Tissue Kit and amplified a 543 pb fragment of the mitochondrial gene cytochrome oxidase I (COI), using specific primers from Lizenberger & Chapco^66^ in 50 µl reactions consisting of: 50 mM Cl2Mg, 50 mM dNTPs, 10 mM of each primer, 50 U/ml Taq polymerase (Invitrogen), 10 X buffer and 100 ng DNA template. After an initial denaturalization at 94 °C for 3 min, we conducted polymerase chain reaction (PCR) via 35 cycles of 94 °C for 30 s, 47 °C for 45 s, and 68 °C for 55 s. The final extension was conducted at 68 °C for 10 min. After visualization on a 1% agarose gel with a 100 bp ladder, PCR products were purified and sequenced on an ABI PrismTM Sequencer 31309 l Genetic Analyzer (Applied Biosystems, Inc.) by the Macrogen INC., Seoul, Korea Sequencing Service.

### Population genetics analysis

Sequences were aligned using CLUSTALX 1.81^67^ and edited using BIOEDIT 7.0.9^68^. The haplotypes were identified and characterized using GENEALEX 6.0 software^69^.

Genetic variation was estimated using haplotype diversity h^70^ and nucleotide diversity π^71^ with the software ARLEQUIN 3.5^72^.

Analysis of molecular variance (AMOVA)^73^ was applied to the dataset to evaluate the hierarchical partitioning of genetic variation among Biomes, populations and individuals using ARLEQUIN 3.5^72^. AMOVASs based on F_ST_ (using haplotypes frequencies) and Ф_ST_ (using genetic distances with Pairwise difference algorithm) were conducted to investigate differentiation among biomes and populations within biomes. The significance of the results was tested through 1000 non-parametric permutations. We used BAPS (Bayesian Analysis of Population Structure) 5.0^74^ to provide deeper insight into the species genetic structure by clustering genetically similar individuals into panmictic groups. BAPS adopts a Bayesian approach with a stochastic optimization algorithm for analyzing models of population structure. The analysis was run with the “mixture of groups of individuals” option. Ten replicate runs for each successive K from 2 to 11 were completed, and the K with the highest posterior probabilities was taken to be the best partition.

### Phylogenetic and demographic analyses

NETWORK 4.6^75^ was used for haplotype network construction. The median-joining method, which combines the features of a maximum-parsimony heuristic algorithm and a minimum spanning tree search algorithm, was used to resolve the relationships of the haplotypes.

To test for evidence of recent demographic expansion, we performed tests of Tajima’s D, Fu’s Fs and mismatch distribution analyses as implemented in DnaSP 5.1^76^. Negative values of D and Fs may be evidence of recent population expansion^77^ although Fu’s Fs is more specifically indicative of population demographic processes^77,78^. The significance was assessed by 1000 permutations. We also generated mismatch distributions, which may provide additional evidence of recent expansion based on the modality of the frequency of pairwise differences between samples. A unimodal shape suggests population expansion, whereas demographically stable populations should display a multimodal distribution. For every distribution, we calculated the Harpending’s raggedness index (r), under the population stability model (r becomes larger the longer populations have been stable)^79,80^.

Aligned sequences were run through the program Mr. MODELTEST 2.3^81^ to estimate an appropriate model of sequence evolution. The best fit sequence model was determined by use of the Akiake Information Criterion (AIC).

Phylogenetic dating and divergence time estimates were evaluated in the program BEAST 1.8.4^82^ by comparing the likelihood of three different models for the coalescent tree prior: constant population size, exponential growth and Bayesian skyline, assuming strict molecular clock and a relaxed uncorrelated lognormal clock for each tree prior, a GTR + I sequence evolution model as given by Mr. MODELTEST and a fixed substitution rate of 1.15 × 10^−2^subs/site/lineage/million years, a rate estimated relative to insects used for COI mitochondrial gene^83^. To test for deviance from a strict clock, we assessed the distribution of the relaxed clock coefficient of variation in TRACER 1.4^84^; if the distribution of variation in branch rates includes zero, the rates are similar enough that a strict clock cannot be rejected^82^.

After verifying that a strict clock can be discarded, a time-calibrated Bayesian tree was reconstructed, through a Bayesian skyline model (constant size and expansion growth models prior failed to reach convergence in multiple analyses), employing an uncorrelated relaxed clock model (lognormal distribution, ucld. mean = 0.0115), previously identified sequences of *D elongatus* as outgroup^85^. For each analysis we conducted two independent runs of 60.000.000 generations, with trees sampled every 6000 generations. We used TRACER 1.4^84^ to determine a burn-in of 10% (6,000,000 generations) and to ensure all parameters of interest in the combined trace files had effective sample sizes (ESS) > 200. Results from replicate runs were pooled with LogCombiner v2.1.2. The analysis was run for 60 million generations and the posterior output was examined in TRACER 1.4^84^ to assess mixing and convergence of MCMC chains. Tree files were summarized with TreeAnnotator 1.7.4^86^ to estimate maximum clade credibility (MCC) tree after removing the burn-in. The final tree was displayed in TreeFig 1.4 (http://tree.bio.ed.ac.uk).

To complement mismatch analyses and estimate timing of demographic events and most recent common ancestor (TMRCA), Bayesian skyline plots (BSP) were implemented in BEAST software, which shows a visual representation of the shape of population change over time. We used the same parameter settings and MCMC diagnostics as our BEAST divergence time analysis using a log-normal relaxed clock with a normally distributed tree height prior corresponding to MRCA for *D. vittatus* (mean = 0.98, SD = 0.2). The analysis consisted of 40 million iterations sampled every 4000 iterations. Bayes factors were calculated by manually summing the tree likelihood and coalescent/skyline columns in the BEAST log file.

### Coalescent simulations

To compare different biogeographic hypotheses on historical migration rates of *D. vittatus*, we used the coalescent-based program MIGRATE-N version 3.7.2^87^. The mutation-scaled population size parameter θ (θ = 2*N*_*f*_μ, where N_*f*_ is the effective population size of females and μ the mutation rate per site per generation), and the pairwise population mutation-scaled migration rates M (M = m_f_/μ, where m_f_ is the female migration rate per generation) were estimated using F_ST_ estimates and a UPGMA tree as starting parameters. The numbers of female migrants per generation 2N_f_m_f_ (equivalent to Nem_f_) were estimated from M and θ.

Analyses were run with five Markov chains and gamma-distributed priors on M (range 0.01–4000) and θ (range 0.0001–0.1). MIGRATE was run 5 times, with the UST-based starting parameters and different random seed numbers. The static heating scheme was set to temperatures 1.0, 1.5, 3.0 and 10,000.0. Studies were conducted using the UST-based starting parameters, with five replicates, with 100,000 steps recorded every 100 generations and a number of discard trees per chain (burn-in) of 10,000 totaling 50 million parameter values retained. For the other settings, we used the default values. All individuals were included and Migrate was run three times with the conditions mentioned above and the run which exhibited higher likelihood score was used to infer historical gene flow. For comparison, the migration rate was also estimated according to F_ST_ = 1/(4 Nm + 1)^88^.

**Figure 4:**
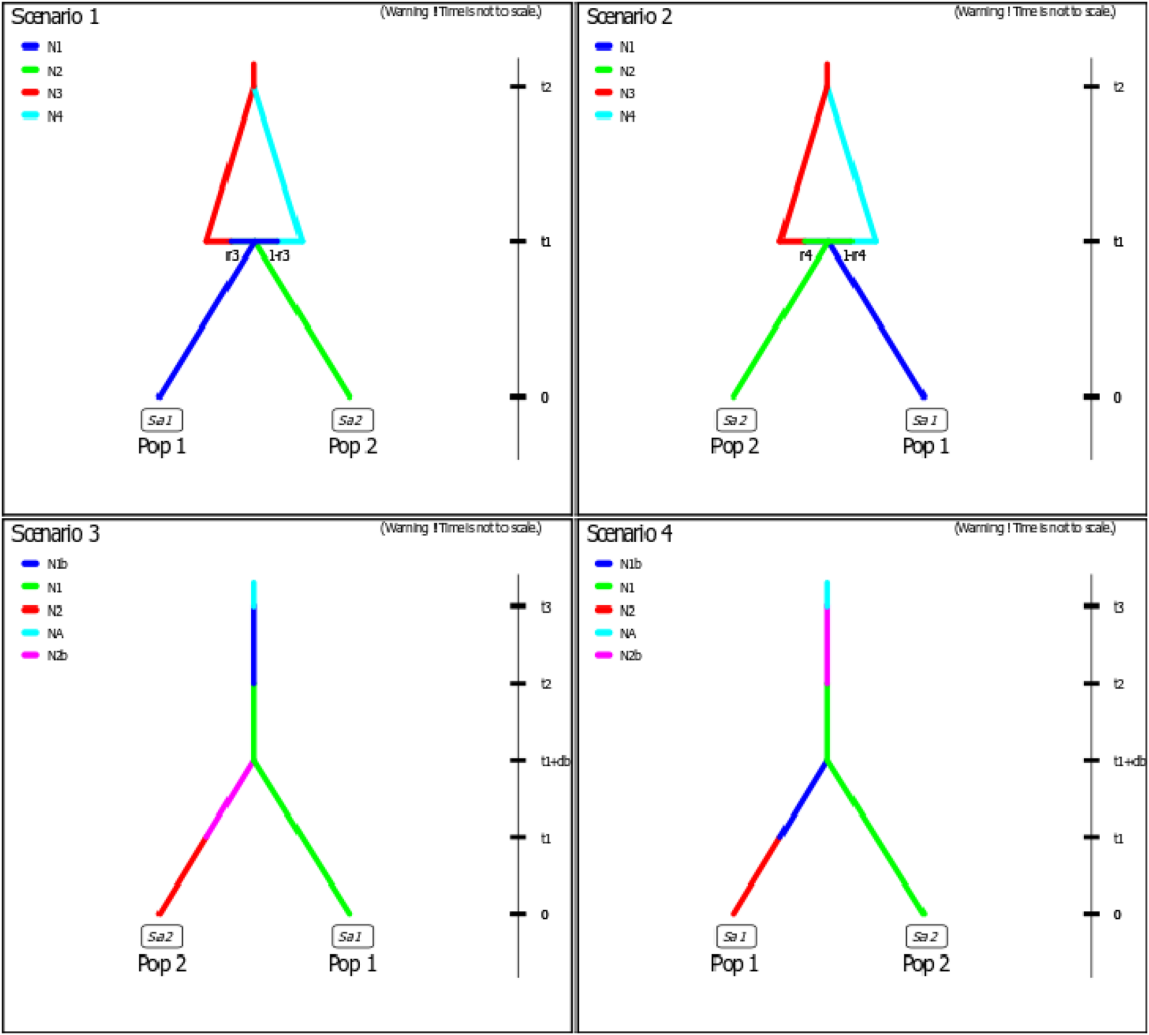
Evolutionary scenarios tested using DIYABC. Scenario 1 assumes that the colonization of both Biomes occurred from a single ancestral population (possible glacial refuge), populations established in Grassland and Savanna diverge and later an admixture event occurs with south- north direction. Scenario 2 proposes a similar demographic hypothesis to Scenario 1, except that the colonization that resulted in the admixture event occurred from north (Savanna) to south (Grassland), Scenarios 3 assumes that colonization occurred from a single ancestral population, reaching the Grassland region first and from there continuing north, reaching Savanna. Scenarios 4 represent the hypothesis of a colonization event with north- south (Savanna- Grassland) direction, from a single ancestral population and the subsequent divergence of the populations established in both Biomes. Changes in effective population size (Ne) are represented as differently shaded lines. Pop1 is referred to Grassland and Pop2 to Savanna.

In addition to traditional population genetics approach we use Approximate Bayesian computation analysis implemented in DIYABC version 2.0 beta^89^ to explore putative scenarios of colonization and divergence in the studied area, posterior to LGM glacial refugia expansion. This approach simulates the data sets for several predefined scenarios and compares the summary statistics of these with the summary statistics of the observed data, making it feasible to test complex population genetic models. Then, statistical tests were used to define which scenario best fits the observed dataset. For this study, we tested 4 different paleodemographic hypotheses that could explain the current genetic structure of *D. vittatus* populations using a fragment of COI gene (Fig. 4). SC1: Both biomes are colonized by individuals from an unsampled ancestral population that could be part of a glacial refuge. Established in the new environments, populations of both Biomes begin to diverge; subsequently an admixture event occurs through gene flow with a South-North direction (Grassland to Savanna). SC2: Proposes the same as the SC1 but the direction of the colonization that results in the admixture event is from North to South (Savanna to Grassland). SC3: The colonization originated from an unsampled ancestral population, and the expansion process occurs from South to North, first reaching Grassland with the consequent increment in Ne and then continuing the colonization route towards Savanna. Finally, the populations established in both Biomes diverge. SC4: The expansion process from an unsampled ancestral population occurs in a North–South direction (from Savanna to Grassland). Therefore, the founder individuals settle first in Savanna and latter in Grassland.

The DIYABC program allows for estimating the posterior probability of the different scenarios compared. Due to the absence of available data for the effective size related parameters (N1, N2, N3, N4, NA: Population size and N1b, N2b: Reduced population size), we used a uniform and broad priors with the limits exceeding the probable smallest and largest population sizes for this species (minimum value for N: 50, maximum value for N: 50,000). The prior distributions for time related parameters were set based in historical references of the Last Glacial Maximum (LGM) in South America^27–29^. We assumed a JK mutation model with a uniform prior distribution of mean mutation rate from 10^−7^ to 10^−2^. Six different summary statistics were considered: number of haplotypes, number of segregating sites, mean of pairwise differences, variance of pairwise differences, Tajima’s D, and private segregating sites. The simulations were repeated 4.000.000 times, and, after logit transformation, local linear regression was applied to choose the 1% simulated data sets that were closest to the observation. We compared the posterior probability of the competing scenarios using a polychotomy weighted logistic regression^90^ with linear discriminant analysis^91^. Confidence in the scenario choice was evaluated by computing type I error (risk to exclude the focal scenario when it is the true one) and type II error (risk to select the focal scenario when it is false) in the selection of scenarios. Posterior model checking was performed on the selected scenario of every analysis using a local linear regression on 1% of the simulated data sets closest to our real data^92^. All summary statistics were used for model checking.

## Supplementary Information

Supplementary Information.
