# Supplementary material for: Evolutionary history and colonization patterns of the wing dimorphic grasshopper Dichroplus vittatus in two Argentinean biomes
Source: Sci Rep. 2022 Feb 21;12:2920. doi: 10.1038/s41598-022-05162-6 (PMC8861051; doi:10.1038/s41598-022-05162-6)
Supplement: Supplementary file 1 — Supplementary Information. [file 41598_2022_5162_MOESM1_ESM.pdf]

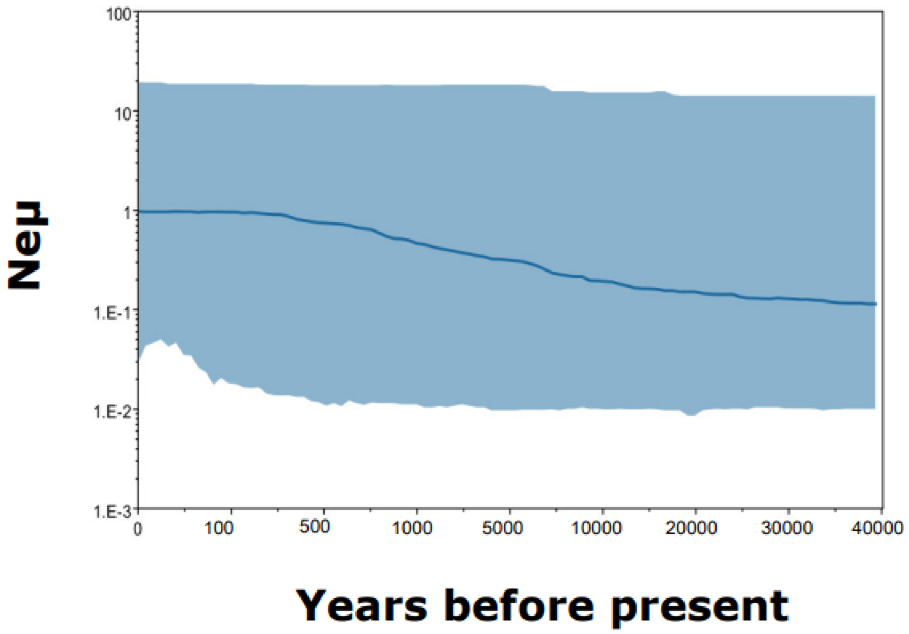

Figure S1: Bayesian skyline plots of the Grassland Biome. The x-axis represents time in units of years and the y-axis represents effective population size as Ne on a log scale. The blue line depicts the median population size, and the shaded areas represent the 95 % highest posterior density intervals.

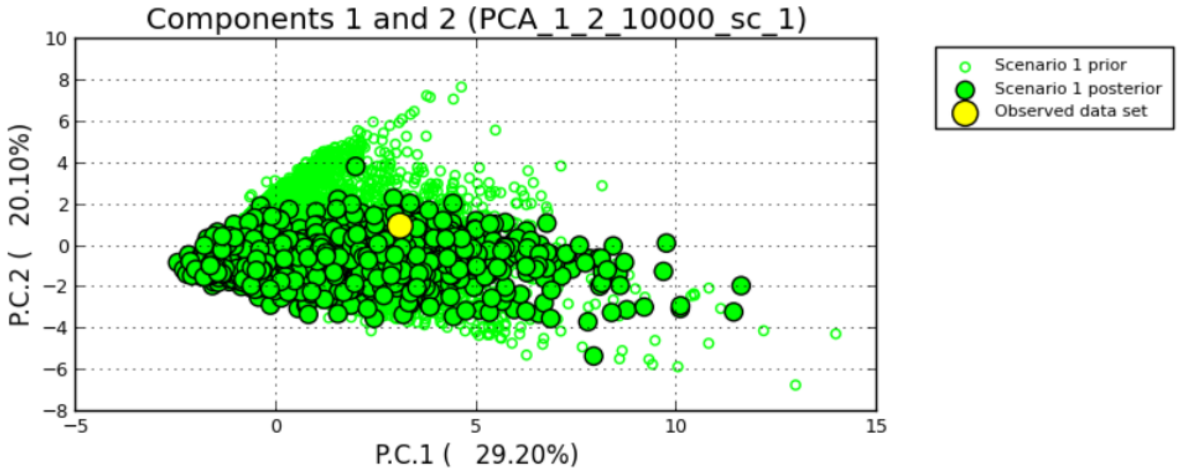

Figure S2: PCA plots for DIYABC model checking. The observed data set (yellow circle) was within the first two axes of PCAs on summary statistics estimated from the posterior predictive distribution of parameters (filled green circle) and simulated prior parameter distribution (open green circle).
